# Rift Valley Fever: Important Considerations for Risk Mitigation and Future Outbreaks

**DOI:** 10.3390/tropicalmed5020089

**Published:** 2020-06-02

**Authors:** Elysse N. Grossi-Soyster, A. Desiree LaBeaud

**Affiliations:** Department of Pediatrics, Division of Infectious Disease, Stanford University School of Medicine, Stanford, CA 94305, USA

**Keywords:** Rift Valley fever (RVF), arboviruses, mosquito-borne viruses, zoonoses, One Health, travel medicine, livestock, viral emergence

## Abstract

Rift Valley fever virus (RVFV) is a zoonotic phlebovirus of the *Phenuiviridae* family with great opportunity for emergence in previously unaffected regions, despite its current geographical limits. Outbreaks of RVFV often infect humans or domesticated animals, such as livestock, concurrently and occur sporadically, ranging from localized outbreaks in villages to multi-country events that spread rapidly. The true burden of Rift Valley fever (RVF) is not well defined due to underreporting, misdiagnosis caused by the broad spectrum of disease presentation, and minimal access for rapid and accurate laboratory confirmation. Severe symptoms may include hemorrhagic fever, loss of vision, psychological impairment or disturbances, and organ failure. Those living in endemic areas and travelers should be aware of the potential for exposure to ongoing outbreaks or interepidemic transmission, and engage in behaviors to minimize exposure risks, as vaccinations in humans are currently unavailable and animal vaccinations are not used routinely or ubiquitously. The lack of vaccines approved for use in humans is concerning, as RVFV has proven to be highly pathogenic in naïve populations, causing severe disease in a large percent of confirmed cases, which could have considerable impact on human health.

## 1. Introduction

Rift Valley fever virus (RVFV) was first isolated in Kenya as a virus with the capacity to infect livestock herds of sheep and cattle, as well as humans [1]. Since its initial discovery, RVFV has been primarily contained within the African continent, with the exception of movement off of the eastern coast of African to the island of Madagascar in 1990 [2,3]. Significant emergence into neighboring regions occurred in the early 2000s when outbreaks were reported in Saudi Arabia and Yemen [4,5,6]. To this day, much of sub-Saharan Africa and Egypt is endemic for RVFV or has been affected by sporadic outbreaks [7,8,9,10,11,12,13,14,15].

Transmission of RVFV utilizes mechanisms described by the “One Health” framework, wherein the health and conditions of the environment, animals, and humans intersect and influence each other. Animal transmission is driven by mosquito vectors, primarily *Culex* spp. and floodwater-breeding *Aedes* spp. [9,16,17,18,19]. Wild animals have been suspected to contribute to maintenance of RVFV, yet evidence driving such speculation is limited to the presence of antibodies in certain wildlife species [20,21,22]. Amplification of the virus in mosquitoes [23,24], is linked to mosquito abundance and breeding behaviors that are expanded by periods of heavy rainfall following extreme drought [9,25,26,27,28,29,30,31]. Of the many competent vector species [17], infected females of some mosquito species may transmit the virus to their offspring during oviposition, or transovarial transmission (TOT) [32], readily allowing future generations of mosquitoes to transmit RVFV [33]. Transmission in livestock is initiated by mosquito bite and amplified within herds by direct contact with infected bodily fluids, yet there has been little evidence of transmission between animals by way of respiratory droplets and nasal discharge that are characteristic of common respiratory infections [25,34]. There is significant evidence to suggest that vertical transmission may be possible in pregnant animals that are not viremic [35], although findings are limited to laboratory studies and cannot confirm viable offspring following in utero exposure, as infection of pregnant animals typically results in abortion storms that eliminate any viable offspring [36]. 

Humans can be exposed by mosquito bite or through contact with infected fluids and tissues. Many studies suggest vector-borne transmission is less likely for humans [34]. Zoonotic exposures are driven by many of the occupational and homestead behaviors that are performed with regularity, such as herding, milking, slaughtering animals, and tending to animal health needs in both veterinary and animal health worker capacities [37,38,39,40]. Occupational exposures have been shown to elicit a higher incidence than individuals having close contact with or caring for animals at the homestead, and is likely related to contact with a higher volume of animals and their fluids [41]. Aerosolization is also a possible, although unlikely route of transmission, and has been correlated with a higher likelihood of severe disease in laboratory experiments [42]. 

Despite the presence of RVFV in Africa and the Middle East, emergence of the virus has the potential to cause catastrophic damage to naïve populations of animals and humans. Competent vector species have been identified in many regions that are currently unaffected by RVFV [43,44,45], providing the ecological support for amplification by mosquito breeding and transovarial transmission (TOT) [32,33]. Rift Valley fever (RVF) causes mild to severe disease in many animal species, with an inverse relationship between the age of the animal and morbidity and mortality, where the younger the animal, the higher the likelihood that the infection will be fatal. Infection in older animals usually produces mild, self-limiting febrile and respiratory symptoms, with a mortality rate ranging from 10% to 30% [46]. Disease severity is also dependent on the species of the animal, and may be specifically virulent in sheep, followed by other commonly domesticated animals such as goats, cattle, buffalo, and camels [45]. While initial symptoms in animals tend to be non-specific, such as diarrhea, vomiting, and respiratory disease, more notable signs of RVFV infection in animals include epistaxis, wasting, spontaneous abortion by pregnant animals, and animal fatalities [25,45]. 

In humans, RVF disease presentation varies widely, and factors contributing to disease severity are widely unknown. Many experience mild, non-specific, and self-limiting febrile illness that may occasionally present as a biphasic fever with an intermittent remission period of 1–2 days between febrile events [47]. More severe symptoms, typically occurring in up to 8–10% of cases [48], include ocular scarring, central nervous system (CNS) involvement, hemorrhagic fever, organ failure, and death [47,49,50]. RVF can also cause human abortions, still births, and congenital infections [51,52,53].

Approximately 1–2% of cases experience hemorrhagic fever symptoms, wherein up to 50% of hemorrhagic cases are fatal [10]. The increased risk of fatality with hemorrhagic presentation may be due to a loss of fluids and multisystem shock, organ failure related to loss of blood volume and fluids, or lack of or mismanagement of symptomatic treatment. In vitro studies have suggested that hemorrhage resulting from RVFV infection may be linked to transcription factor IIH (TFIIH) expression levels [54], yet there have yet to be effective treatments for viral hemorrhagic fevers (VHF) beyond basic symptomatic treatment and monitoring [48]. It has been suggested that hemorrhagic cases of RVFV infection may increase the risk of nosocomial transmission for healthcare workers and other individuals providing care [55], yet human-to-human transmission by nosocomial routes of exposure have yet to be documented.

Despite RVF commonly being presented as a mild, self-resolving febrile illness, disease severity has varied by region in epidemiological reports. Publications from Yemen from January 2014 to August 2016 reported hemorrhagic fever in 9% of their anti-RVFV IgM positive hospitalized patients [56], whereas estimates for hemorrhagic symptoms are often limited to 1–2% of cases [57,58]. Early outbreaks in Saudi Arabia experienced approximately double the amount of fatal cases than neighboring Yemen [4], which is likely due to insufficient immunity in the previously naïve community, or increased pathogenicity and disease severity as a result of genomic mutations and reassortments [59]. Variability in disease severity is also seen in neighboring regions, such as countries in East Africa, or intercontinental differences seen in Egypt versus in countries in sub-Saharan Africa and the horn of Africa that are affected by RVFV [7].

Ocular scarring is often reported in 10% of patients [50], while some outbreaks have been associated with more than 40% of patients experiencing loss of vision [60]. Patients experiencing ocular symptoms typically report blurred or loss of vision and posterior eye pain, possibly caused by the development of lesions, edema at the optic disc, or retinal vasculitis or hemorrhaging [49,59,60]. Loss of vision as a result of RVFV infection may be temporary or permanent, depending on the location and severity of the lesions within the ocular tunics. Reports have not distinguished a unilateral or bilateral effect specifically associated with RVFV infection, as confirmed RVFV-positive patients have been documented to suffer retinal scars both unilaterally and bilaterally [49,59].

Multisystem effects of acute RVF are illustrated by involvement of the liver and kidneys, occasionally leading to the onset of hepatitis and nephropathy [34,48,60]. Jaundice and splenomegaly are commonly found in patients during physical exams for diagnosis, and should be monitored carefully to avoid progression to multiple organ failure [48].

Many studies have attempted to identify mechanisms of neurological complications from RVF, yet clear pathways, even those suggesting immune-mediation, have yet to be identified [61,62,63]. CNS involvement may superficially appear as dizziness or vertigo, confusion and disorientation, and intense headaches, yet may suggest severe underlying manifestations. Meningoencephalopathy can occur in 1–2% of cases and may lead to convulsions, coma, or death [34,60,61,62,64]. Psychological evaluations of such symptoms suggest CNS involvement may elicit the onset of mental health syndromes, with diagnoses similar to schizophrenia [65,66,67], and should be taken into consideration when considering immediate treatment and care options. Patients with progression of such syndromes should be evaluated for long term sequelae, as the persistence of psychological syndromes related to RVFV infection has yet to be fully described. 

Inconsistent prevalence and incidence of RVFV infection reported is possibly linked to untimely reporting or underreporting of cases or lack of laboratory confirmation in cases of suspected diagnosis [7]. Acute cases are best confirmed by reverse transcriptase polymerase chain reaction (RT-PCR) [68,69,70], but facilities equipped with the resources, such as skills and instrumentation, required for thorough diagnosis using PCR may be sparse in many endemic regions. Epidemiological studies for assessing RVF burden are often limited to community surveys based on serological analysis of retrospective infection, represented by the presence of immunoglobulin G (IgG) antibodies [68,71]. Detection of immunoglobulin M (IgM) antibodies may be possible [70,71], yet assays designed for IgM detection are notoriously problematic, with potential cross-reactivity and interfering factors (such as rheumatoid factor) leading to inconsistent results, and are therefore not as reliable as PCR diagnostics for acute cases. These analyses may not describe the true burden of RVF in a given population, as acute infections are rarely detected and clinical factors cannot be monitored in real time. 

Underreporting may also be due to stigma associated with reporting cases of RVF in animals and humans, which is a phenomenon that is not limited to RVF, but described broadly with infectious diseases throughout history [72,73]. Stigma against RVF survivors has not been reported [38], yet both internal and societal stigmas borne from restrictions with livestock trade and sales may influence downstream behaviors. Trade restrictions for three years are implemented when animal cases are reported and confirmed [74], which may have a major impact on local economies and personal incomes [75]. Additionally, animal infections can trigger a loss of revenue from a reduction in herd size from livestock deaths, and delayed production of sellable animal products due to illness and costly quarantine procedures [75,76]. Spontaneous abortion in pregnant animals also reduces future product generation capacity with the loss of offspring, influencing further financial burden. Community beliefs about processing animal carcasses and use of specific animal parts after death drive personal behaviors that are negligent of the estimated risk of disease exposure, leading individuals to continue engaging in behaviors to avoid bad luck or cultural stigma despite increased risk of personal exposure or continued exposure of other animals, such as skinning animal carcasses before disposal, or harvesting and/or consuming specific organs [77]. Appropriate risk-mitigating behaviors may not be engaged if the perceived risk of exposure is low, or not well understood.

Travel and tourism catalyzes new opportunities for infections in international populations, with the ease of air travel allowing for acutely infected individuals to rapidly reach new destinations [78]. In 2010, Germany had a suspected case of RVF after a tourist visited South Africa, initiating travel warnings for FIFA World Cup events [79,80]. Other regions are suspected to be sources of potential imported cases to Europe, such as Dakar and neighboring regions in Senegal, as they are popular tourist destinations for European residents [81,82]. More recently, an acutely ill individual with persistent symptoms traveled to China after working in Angola in 2016 [83,84,85]. Traveler-acquired cases of RVF continue to occur, and the public health implications of such cases continue to stress the importance of accurate incidence and prevalence reporting, rapid diagnostic availability and affordability, and the need for a vaccine for human use.

## 2. Vaccines

RVFV is comprised of a negative-sense, single stranded RNA genome cleaved into three segments varying in size. The functional strength of the genome is driven by the encoding of four structural proteins and two nonstructural proteins. While much is understood about the RVFV genome and viral replication, the genome also contains a sequence for a 78-kDa protein, called Large glycoprotein (LGp) [68,86], which may contribute to viral dissemination in vectors [87,88].

All of the current vaccines licensed for use in animals are generated using viral strains from early outbreaks and isolates, ranging from 1948 to 1977 [68]. Serial passaging has proven some vaccine strains to maintain genomic stability [89], showing minimal risk of reversion to original strain virulence with continual generation and use of the vaccines, but there may be an increased risk of incomplete protection or coverage from newly evolving wildtype strains in circulation. Vaccines designed from early strains do not accommodate the lineage diversity, and may not effectively continue to protect as mutations and reassortments are introduced [58].

Vaccines approved and licensed for use in non-endemic countries are nonexistent, with the exception of MP-12, that has conditional licensure in the US. MP-12 is a live attenuated strain produced by repeat passaging of Egyptian wild type (WT) strain ZH548 in the presence of 5-Fluorouracil, a mutagen that inhibits RNA processing and DNA synthesis [89,90,91]. Initial viral challenge studies in livestock showed few side effects, with the exception of mild liver necrosis in calves resulting from high dosage administration [92], but many farmers and herders that have used the vaccine have reported low levels of spontaneous abortion when MP-12 is administered to pregnant animals [46,92,93]. Variations on the original MP-12 attenuated vaccination, such as deleting nonstructural genes from the attenuated strain, have also been successful at preventing disease and minimizing lethality, but have yet to be thoroughly tested in the field [94,95,96,97,98,99]. MP-12 was derived using a human WT strain, and has been proven to be safe for use in humans [100,101], but the duration of neutralizing antibodies has yet to be established, therefore a vaccination schedule has not been proposed. Novel vaccines designed with recombinant proteins, virus-like particles and replicons, and live virus vector-based vaccines which carry DNA-encoding antigens for RVFV in viral vectors such as poxviruses and adenoviruses, have been evaluated for their performance in animal models, such as mice, sheep, and cattle, but have yet to be evaluated for human performance [86,102,103]. Thorough reviews of each of these advanced vaccine designs have been recently summarized [68,86]. 

At the start of 2019, The Coalition for Epidemic Preparedness Innovations (CEPI) released a call for the development of a human vaccine against RVFV [104], due to RVFV’s inclusion on the priority pathogens list released by the World Health Organization [105,106]. CEPI’s decision to invest in RVFV was based on the “feasibility of vaccine development and the potential public health impact,” [104], which is undoubtedly influenced by the groundwork laid by prior vaccine development for animal use, the increasing frequency of RVFV outbreaks in the last decade, and growing concerns about potential emergence into new territories, such as the United States and the European Union [43,44,68]. Many may question the prioritization of the development of a human vaccine against RVFV, as the mortality rate is reportedly low compared to other, more pressing infections, yet recent viral emergence and extensive outbreaks for which effective vaccines and therapeutics were unavailable, seen with Zika virus (ZIKV) in the Americas and Ebola virus (EBOV) outbreaks in Africa, have shown the need for vaccine development and approval prior to emergent events. The sudden emergence into the Middle East illustrated RVFV’s capability to migrate to previously unaffected areas facilitated by human and economic mobility, and the differences in disease severity when previously naïve populations are exposed [4,5,6,37,56,59,107]. There are currently two RVFV vaccines for humans in Phase II clinical trials [106], with nearly two dozen other vaccine candidates in the preclinical phase of development [106]. If approved, vaccines in humans could be produced for emergency use, to minimize the spread of future human outbreaks within sub-Saharan Africa and the Middle East, or selectively used in individuals with an increased risk of exposure, as the rabies vaccine is given to those with occupational exposure or travel risks. A human RVFV vaccine should also be used to mitigate interepidemic incidence in currently impacted regions. If a safe and effective RVFV vaccine for human use is administered in conjunction with animal vaccines and thorough public health education efforts regarding disease awareness and risk mitigation behaviors, RVFV may be less likely to be considered a priority pathogen in the future.

As novel vaccines are designed and evaluated, it is important to consider the goal of each of the vaccine candidates. Given the complexity of the RVFV transmission cycle, will a vaccine ever effectively prevent RVFV outbreaks in animals and humans, or will the aim of vaccine programs be to minimize disease and fatalities? Further, is it likely that one vaccine will be able to establish immunity in animals and humans, whether in impacted countries or in currently unaffected geographical regions? Future iterations of animal vaccines should be designed for prioritized administration in younger animals, and further vaccines should be evaluated in pregnant animals. RVF-linked abortion has also been reported in humans [51,52,53], yet currently available vaccines have not been evaluated for their performance in conferring neutralizing antibodies while also avoiding harmful effects on fetal development and viability.

Designs of future vaccine candidates should also consider the economic impact on individuals who will require the vaccine. Vaccines should aim to be economical with minimal burden for cyclical vaccination schedules and boosters. Vaccine workshops in 2011 established that novel vaccines should confer long-duration immunity after a single dosage [102], which has yet to be established with current vaccine iterations that require repeated and seasonal vaccines to induce neutralizing antibodies [94]. Vaccine programs would benefit from vaccines that are designed with the ability to differentiate infected animals from vaccinated animals (DIVA), as many of the current vaccine candidates fail to meet DIVA standards [86]. The ability to effectively differentiate animals that have been naturally infected from those who have been preventatively vaccinated is vital in mounting public health responses to impending and active outbreaks, especially in non-endemic populations. DIVA is also essential to promoting the World Organisation for Animal Health prevention requirements, which precludes any vulnerable animal importation from countries considered to have been infected by RVFV within the last 3 years. Vaccines should also consider the severity and duration of side effects that may impact an animal’s ability to produce milks and other products. Farmers, herders, and individuals that maintain livestock herds will be potentially less likely to use a vaccine that produces side effects or physiologically impacts their animals in a way that would impact their production and income.

Vaccination programs aiming to minimize disease severity will require further investigation into the immunopathophysiology of RVFV and identify host immune mechanisms that may increase the likelihood of severe disease symptoms. Severe disease appears to be more often experienced in populations without prior exposure to RVFV, as seen with the emergence into the Arabian Peninsula [4,5,56,59,107], and without further knowledge of causative agents of severe disease, currently naïve populations should be assumed to be at a higher risk for severe morbidity and increased rates of mortality. As witnessed with recent emergence of ZIKV in the Americas [108,109], the impact of unanticipated viral emergence in a novel geographical region or human population can have catastrophic effects. 

## 3. Public Health

Outbreaks continue to occur sporadically, yet their frequency has increased in the last decade, and larger populations are commonly affected. Most recently, an outbreak originating in South Sudan [110] was unable to effectively be contained and may have grown to affect multiple countries across southern and eastern Africa, including Kenya, Rwanda, Uganda, and South Africa [111,112,113,114], although some suggest that the outbreaks in each country were not related. Public health measures regarding RVF often focus towards avoiding outbreaks or minimizing the perpetuation of outbreaks rather than minimizing individual exposures and prophylactic measures. Many reports stress the importance of vaccination as the leading way to control outbreaks, yet usage of available vaccines is not standardized or enforced across all endemic regions [7,34,57,74,86]. Additionally, livestock trade between villages and bordering countries may make it difficult to monitor vaccine administration and to achieve effective herd immunity rates to control widespread infection. In addition to aggressive vaccination campaigns, public education is imperative to mitigating continual zoonotic exposures [77].

In naïve regions, concerns regarding use of RVFV as a bioterrorism agent are heightened by the spectrum of disease in animals and humans, and for the potential to devastate large-scale agricultural economies. RVFV is considered a Category A pathogen in the Center for Disease Control and Prevention (CDC)’s Bioterrorism Agent/Disease classifications [115], and as an overlap select agent by the Health and Human Services (HHS) and United States Department of Agriculture (USDA) Federal Select Agents Program [116]. The United States, the Netherlands, the United Kingdom, and many locations in the European Union have been established as potential points for emergence of RVFV in the future due to the availability of mosquito species that are capable of transmitting RVFV, extensive livestock economies and trade, and potential wildlife hosts for interepidemic maintenance [43,44,117,118]. Changes in climate and seasonal extremes may expand the potential for introduction of RVFV to these areas, supporting expansion of vector abundance and conditions for livestock [31]. Air travel contributes to the rapid transport of imported cases, which has been a leading catalyst of the emergence of many arboviral diseases, such as Zika virus and chikungunya virus, that are now established and autochthonously transmitted in previously unaffected areas [57,78,108,109]. While human-to-mosquito transmission is currently speculative for RVFV, many other supportive factors are in place to enhance the likelihood of an emergent outbreak of RVFV.

## 4. Current Risks and Considerations for Travelers

Travelers should take a specific interest in the risks and exposure opportunities in much of Africa and the Arabian Peninsula. A multifaceted approach to risk mitigation will dramatically reduce the risk of infection, and should include mosquito avoidance, safe practices with animals and animal products, and avoiding contact with infected fluids, tissues, and potential avenues for aerosolization.

Mosquito repellent should be used thoroughly and clothing should be treated to minimize vector biting [119]. *Culex* spp. and *Aedes* spp. that can transmit RVFV are day-biting species, with peaks of feeding near dusk and dawn. Bed nets are an important tool to avoid malaria infection, but mosquito avoidance behaviors should also be practiced during the day for thorough disease avoidance. Mosquito populations in and around the homestead and areas frequently visited can be reduced by dumping out containers that collect standing water, such as tires and buckets, and using air conditioners and screens on windows and doors can dramatically reduce the instance of mosquito exposure. Travelers should also be aware that spraying to control mosquito populations may occur inconsistently, and is often performed in response to an ongoing outbreak, therefore immediate precautions such as the use of personal repellents should be prioritized.

If a traveler anticipates to have contact with animals, specifically domesticated livestock, safe and sanitary measures should be observed in order to reduce contact with potentially infected materials, such as animal bodily fluids and tissues [120,121]. Contact with animals that appear ill or are currently quarantined should be avoided. Consumption of animal products should be limited to items that have been thoroughly cooked to kill any pathogens, including RVFV, as the consumption of meats and milks have been linked to an increased risk of RVFV infection [40,122]. RVFV has the potential to be transmitted by aerosolization [42,49]. Aerosolization is generally a risk observed in occupational settings, such as slaughterhouses, where animal bodily fluids may be aerosolized during processing and handling [41]. Precautions during animal exams, handling animals for milking or other care and maintenance behaviors, and slaughtering or breaking down carcasses should be observed to avoid contact with fluids and blood. 

## 5. Conclusions

RVFV is a complex virus with many possible transmission routes connecting animals and humans, and a wide spectrum of disease without targeted treatment options outside of symptomatic support. RVFV is geographically limited, but many countries contain competent vector species and susceptible hosts that could lead to emergent outbreaks, making RVFV a candidate for an extensive public health burden. Current vaccines are unavailable for humans, which means those living in endemic regions and travelers should practice risk-mitigating behaviors rigorously, and maintain an awareness of the possibility of outbreaks and interepidemic exposure in much of Africa, Saudi Arabia, and Yemen. It is highly encouraged that vaccine development takes into consideration the specific goals of disease prevention, whether future vaccines are utilized to minimize disease or reduce overall incidence.
